# Manually scanned single fiber optical coherence tomography for skin cancer characterization

**DOI:** 10.1038/s41598-021-95118-z

**Published:** 2021-07-30

**Authors:** Nadiya Chuchvara, Babar Rao, Xuan Liu

**Affiliations:** 1 Center for Dermatology, Rutgers Robert Wood Johnson Medical School, 1 Worlds Fair Drive, Somerset, NJ 08873 USA; 2 Rao Dermatology, 95 First Avenue, Atlantic Highlands, NJ 07716 USA; 3 Department of Dermatology, Weill Cornell Medicine, 1305 York Ave 9th Floor, New York, NY 10021 USA; 4 Department of Electrical and Computer Engineering, New Jersey Institute of Technology, University Heights, Newark, NJ 07102 USA

**Keywords:** Cancer, Optics and photonics, Medical imaging

## Abstract

Optical coherence tomography (OCT) is a cross-sectional imaging modality based on low coherence light interferometry. Within dermatology, it has found applications for in vivo diagnostic imaging purposes, as well as to guide Mohs micrographic surgery (MMS), due to its ability to visualize skin morphology up to several millimeters in depth. However, standard OCT probes have a large footprint and capture an extended area of the skin, making it difficult to precisely pinpoint clinically relevant location being imaged. Mohs surgeons stand to benefit from a handheld in vivo imaging device that can accurately trace surgical margins. In this study, we demonstrate the use of a single fiber OCT (sfOCT) instrument. Our imaging system features a miniature common path single fiber probe, and a novel speckle decorrelation technique that generates distortion free 2D images from manual scanning.By manually moving the single-fiber probe across the region of interest, the user can perform a lateral OCT scan while visualizing the location of the probe during data acquisition. Using the sfOCT, we have identified normal skin morphology, qualitatively correlated features of basal cell carcinoma and squamous cell carcinoma with histopathology, and quantified the disruption of the dermo-epidermal junction OCT pattern in skin tumors—each demonstrating the potential of utilizing sfOCT to differentiate tumor from normal skin. Using this imaging tool, a Mohs surgeon can enhance determination of surgical margins for the first stage of MMS, potentially decreasing the time and number of stages required for complete tumor removal.

## Introduction

Nonmelanoma skin cancers (NMSCs), particularly basal cell carcinoma (BCC) and squamous cell carcinoma (SCC), are the most common cancers in the United States^1–3^. On cosmetically sensitive areas such as the face, NMSCs are often managed by Mohs micrographic surgery (MMS). In this procedure, a dermatologic surgeon conducts a stepwise removal of a biopsy-proven tumor, checking for microscopic margin clearance after each subsequent stage by examining histopathological tissue sections which are prepared in-house. In this manner, the surgeon can assure complete removal of the tumor, while minimizing the amount of healthy skin removed. However, multiple stages of tissue excision, histology preparation, and microscopic examination can keep a patient in the office for hours. A cross-sectional, in vivo imaging modality that determines the microscopic boundary of the tumor in real time will increase the precision of the first tissue excision, decrease the number of stages required to accomplish complete removal of the tumor, and shorten the duration of the MMS procedure.

Reflectance confocal microscopy (RCM) offers cellular resolution and has been adopted in clinical dermatology for in vivo diagnosis and tumor margin determination^4^. However, the imaging range of RCM is limited by the depth that its 830 nm laser source can penetrate (200 to 300 $\upmu$m). The small imaging depth of RCM decreases its sensitivity for BCC detection, because BCC can infiltrate deeper than the superficial dermis. The small imaging depth of RCM also limits its sensitivity for SCC detection, as SCC may produce a hypertrophic surface scale that limits the depth of epidermal and dermal features visible on RCM^5–7^. Optical coherence tomography (OCT) is a cross sectional imaging modality with a wide range of biomedical applications^8^. Compared to RCM, OCT offers the benefit of an extended imaging depth (up to several millimeters), and its application for imaging NMSCs has been validated in previous studies^9–11^. However, when determining tumor margins, both imaging modalities (RCM and OCT) use a probe that has mechanical beam scanners to acquire 2D or 3D imaging data and a bulk objective lens to collect signal photons. The probe inevitably blocks the line of sight to visualize the skin tumor during image acquisition. In addition, both RCM and OCT capture an extended area of the skin. For example, Schwartz et al compared RCM and OCT imaging for dermatology, with an 8 mm x 8 mm field of view for RCM and a 6 mm x 6 mm field of view for conventional OCT^12^. Therefore, it is difficult to pinpoint the exact location being imaged. Hence it relies on makeshift landmarks such as ink demarcation, to correlate signal at a specific location in the coordinate of the digital image with a physical location^13^. It is possible to precisely determine the boundary of skin tumor by analyzing OCT images. However, these critical spatial locations (tumor margin) are defined in the coordinate system of the digital image. Currently, there lacks a mechanism to correlate the tumor edge detected in an OCT image with the precise physical location at the patient’s skin.

In this study, we investigated a manually scanned single fiber OCT (sfOCT) instrument—the interface of which is not much larger than the tip of a ballpoint pen—for the delineation of biopsy-proven BCC and SCC tumor margins in patients receiving MMS. Our sfOCT represents a new class of imaging instrument for skin cancer imaging and is significantly different from existing implementations of OCT technology. The sfOCT imaging system is based on a swept source OCT engine, a manually scanned single fiber probe. This system is highly innovative, in its use of a single mode fiber as a common path OCT probe and its unique speckle decorrelation strategy to correct distortion artifacts due to manual scanning^14–17^. Compared to all the other OCT technologies utilized in dermatology, to the best of our knowledge, our sfOCT system has the simplest imaging probe and scanning mechanism. The handheld fiber-optic probe of sfOCT can be used to image cutaneous tumors at virtually any anatomical site with arbitrary lateral dimension. By sweeping the handheld fiber-optic probe perpendicular to the surface of the skin, thanks to the thin imaging probe, the user performs a lateral OCT scan that precisely correlates to the location, which is a significant advantage of using sfOCT probe. In this manner, we obtained OCT images showing different characteristics for BCC, SCC, and normal skin. Our results show the effectiveness of the sfOCT system in skin cancer characterization despite its extremely simple configuration our, including: (1) signals obtained through manual scanning using the single fiber OCT probe showed different characteristics for tumor and normal skin; (2) OCT features acquired by manually scanning at positive margin could be correlated with histopathological features; (3) the dermal-epidermal junction (DEJ) could be identified from OCT signals of the normal skin while skin tumor resulted in disruption of the DEJ. By visualizing the location of the thin OCT probe, the dermatologist can correlate the OCT signals to their location on the skin, to achieve more precise tissue excision in MMS. In summary, this study demonstrated the potential of sfOCT as a simple and effective instrument for skin cancer imaging and tumor margin delineation.

Many studies have shown that both OCT and confocal microscopy can noninvasively detect superficial and nodular BCCs with sensitivities and specificities in the range 80-95% and 70-90%, while several studies show OCT has limited sensitivity and specificity in diagnosing NMSC lesions^18–25^. The focus of our technology is not on diagnosis. Instead, we intend to use the imaging device to identify the boundary of a lesion that has been confirmed as tumor. The thin needle allows precise correlation of OCT findings and anatomical locations. Biomarkers used to differentiate normal skin and diseased skin include disrupted epidermis, plug-like structures and upper dermis signal-free cavities, highly reflective surface with discrete bright regions below the surface, dark nodular structures with a thin, dark border, keratin pearls of SCC, etc. In addition to these biomarkers identified through visual inspection, features extracted through deep learning analysis of OCT image can also be used for tissue differentiation.

## Imaging system

**Figure 1:**
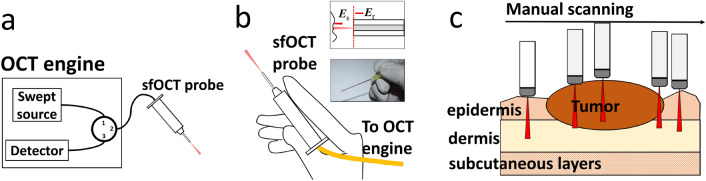
(**a**) Swept source OCT system; (**b**) handheld sfOCT probe (upper inset: reference light derives from the probe tip; lower inset: a photo of the probe held by hand); (**c**) sfOCT probe is manually scanned across a region of interest to generate a 2D image.

The sfOCT system is based on a 1060nm swept source OCT engine (AXSUN). The imaging capability of the swept source engine was validated in our previous study^26^. The output of the swept source is routed by a fiber optic circulator to a single fiber probe (Fig. 1a). We make the single fiber probe (Fig. 1b) by splicing a FC/APC single mode patch cable to a segment of bare fiber, integrating the distal tip of the bare fiber with a needle, and attaching the single fiber probe with a plastic handle. We cleave the tip of the bare fiber to generate a flat surface (upper inset of Fig. 1b). Through Fresnel reflection, the tip of the fiber probe provides a reference light (***E***r). The fiber-optic probe also collects signal photons from the sample (***E***s). In the common path interferometer, ***E***r and ***E***s share the same probe path, and interfere to extract depth resolved information from the sample^14,15^. Unlike a conventional OCT imaging system, the sfOCT system performs lateral scanning by manually steering the probe across a region of interest (Fig. 1c). Therefore, the imaging probe is extremely simple, lightweight and low cost. The fiber-optic probe was integrated with a stainless steel needle (20 gauge feed needle, Roboz Surgical Instrument) that provided rigidity. The needle had a rubber cap at its tip. The fiber optic probe was recessed from the outmost edge of the rubber cap. Therefore, when the rubber cap was in touch with the sample, there remained an offset between the end facet of the fiber and the sample surface. During scanning, the probe gently touched the surface of the skin. The rubber cap ensured a relatively constant pressure between the probe and the skin, and minimized the deformation of skin layers during scanning. It is noted that OCT signals directly obtained from manual scanning suffer from distortion artifacts. The OCT engine acquires Ascans at a constant temporal sampling rate. When the probe is manually scanned, the resultant image has a nonconstant spatial sampling rate induced by the nonconstant scanning speed. To address this issue, we utilize a motion tracking method based on speckle decorrelation analysis, to quantify the lateral displacement between adjacent Ascans and correct distortion artifact^16,17^. Briefly, we calculate the cross-correlation $\rho _i$ between sequentially acquired Ascans ($S_i$ and $S_{i+1}$): $\rho _i=\frac{(S_i-<S_i>)(S_{i+1}-<S_{i+1}>)}{\sigma _i\sigma _{i+1}}$, use the cross-correlation coefficient to quantify lateral displacement: $\delta x_i=w_0\sqrt{ln{\frac{1}{\rho _i}}}$ , calculate the accumulated lateral displacement: $\delta x_n=\sum _{i=1}^{n}\delta x_i$, and sample an Ascan when $x_n$ reaches $\delta x$ that is the pre-determined lateral sampling interval. With the above described method for motion tracking and Ascan resampling, we are able to reconstruct distortion free OCT images using data obtained from manual scanning. The system acquires OCT signal using a frame grabber (PCIe-1433, National Instrument), processes signal in real-time using a GPU (NVIDIA gtx1080), and uses a Dell Precision workstation to coordinate data acquisition, processing and display.

## Imaging experiments

**Figure 2:**
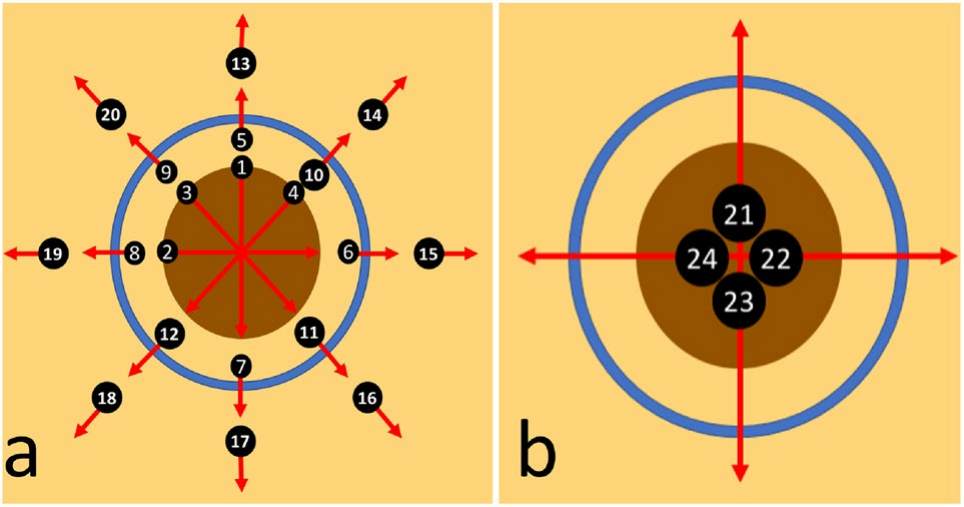
Radial scans performed using the sfOCT along (**a**) clinically apparent tumor (1–4), marked surgical margin (5–12), adjacent normal skin (13–20), and (**b**) a combination of the three (21–24).

To demonstrate the capability of sfOCT in skin tumor delineation, we performed an imaging study on skin cancer patients. Patients who were scheduled to receive MMS for a biopsy-proven BCC or SCC tumor were recruited at a private dermatology practice under institutional review board (Advarra) approved protocol, along with written informed consent and HIPAA authorization. All methods were performed in accordance with the relevant guidelines and regulations. Data obtained was de-identified to protect patient privacy. Prior to imaging, the Mohs surgeon (BR) used a medical-grade marking pen to draw the surgical margin along which the first stage would be excised. Then, for each tumor, 2D images were obtained radially with uniform interval from the center of the tumor through manual scanning following a predetermined protocol. To facilitate retrospective analysis, the scans were taken along trajectories as shown in Fig. 2a,b, across clinically apparent tumor (scans 1–4), marked surgical margin (scans 5–12), adjacent normal skin (scans 13–20), and a combination of the three (scans 21–24). Scans 1–20 were performed with a $17\,\upmu {\text{m}}$ transverse sampling interval and scans 21–24 were performed with a $51\,\upmu {\text{m}}$ transverse sampling interval to cover a larger field of view. Afterwards, the MMS proceeded as usual according to the surgeon’s initial marked margin. We compared OCT signals from tumor and surrounding normal skin, correlated OCT features with histopathological features at positive margin, and identified the DEJ to differentiate tumor and normal skin tissues. Mohs histology sections from the first stage were reviewed and retrospectively compared to the acquired Ascans for correlation of OCT with histopathology. It is not easy to track the path of manual scanning. To be able to correlate OCT scans with histology examination, patterns in Fig. 2 were rigorously followed in our experiments. The scanning process does not take a long time and is usually accomplished within 3 minutes, and there is a tradeoff between scanning time and sampling density. Nevertheless, our scanning protocol allowed fast profiling of lesion boundary, with sampling interval that has a scale manageable by manual scanning and can be correlated with histology.

## Results

**Figure 3:**
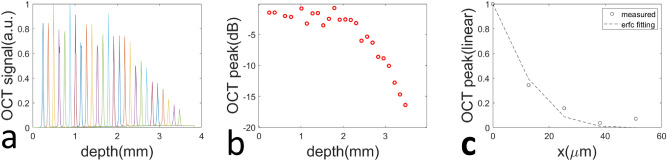
(**a**) Ascans obtained from a mirror at different imaging depths; (**b**) peak signal magnitude for Ascans obtained from different depths; (**c**) lateral profile obtained when the probe was scanned across a specular surface with step reflectivity profile.

We evaluated the performance of OCT imaging based on a lens-less bare fiber probe and demonstrated its capability to provide high quality images for tissue characterization. The beam output from the fiber probe diverges as it propagates, resulting in reduced signal magnitude and lateral resolution at large imaging depth. To characterize beam divergence induced signal attenuation, we obtained Ascans from a mirror (impulse reflective profile) at different depths (Fig. 3a) and show the amplitude of signal peak at different depths in Fig. 3b. The attenuation of signal magnitude remains small (< 5 dB) up to 2 mm in depth, suggesting a sufficiently large imaging depth despite the use of a lensless fiber probe. The axial resolution is currently governed by spectral/spatial domain sampling and was measured to be 6 $\upmu {\text{m}}$ by fitting the axial point spread function (PSF) with a Gaussian. To characterize the lateral resolution of the fiber probe, we scanned the probe across a specular sample that has a step function reflectivity profile and the resultant lateral profile (Fig. 3c) could be modeled as an erfc function, assuming the lateral PSF of the sfOCT system to be Gaussian^27^. Through non-linear least square fitting, we extracted the full width maximum of Gaussian PSF and estimated the lateral resolution to be $35\,\upmu {\text{m}}$ ($w_{0}=\frac{FWHM}{2ln2}$) at the depth of 1.1mm. This is comparable with a conventional OCT system that uses a low NA lens in the sample arm.

**Figure 4:**
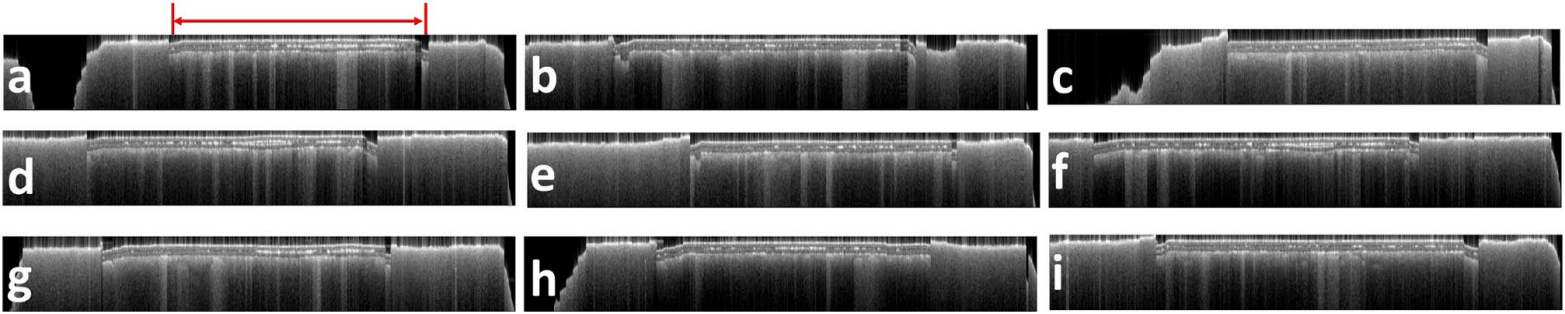
2D sfOCT images obtained by manually scanning the probe across a phantom. The region of interest corresponds to area covered by two layers of tape, as highlighted in (**a**).

The capability of a manually scanned common path OCT probe for anatomically accurate imaging was validated in our previous studies^16,17^. Here we validated the effectiveness of speckle decorrelation analysis in correcting distortion artifacts. We created an imaging phantom by embedding two layers of scotch tape (10 mm in width) into a soft clay substrate. In multiple testing experiments, the sfOCT probe was manually scanned across the phantom. Figure 4 shows 2D images obtained through manual scanning. The images had very flat surfaces, because the probe had a rubber cap and the probe was gently in touch with the sample surface. With speckle decorrelation analysis, the reconstructed images are free of visually apparent distortion artifacts. Moreover, regions of interest (ROI) corresponding to tape layers (indicated by the red arrow in Fig. 4a) have consistent width, suggesting the capability of speckle decorrelation analysis to reconstruct structurally accurate image through manual scanning. Using the number of Ascans within the ROIs and the known width of the tape, we estimated the lateral displacement between adjacent Ascans to be 17.4 $\upmu {\text{m}}$ this is consistent with the sampling interval we specified in the software ($\delta l=18\,\upmu {\text{m}}$).

**Figure 5:**
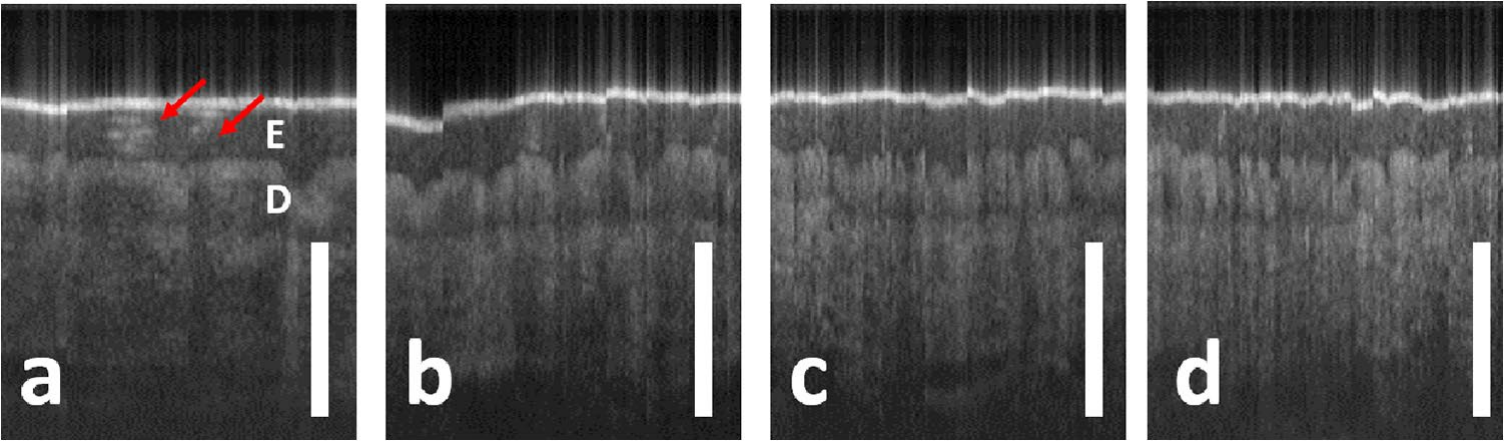
2D sfOCT images obtained by manually scanning the probe the skin at the index finger, at different lateral sampling intervals. $\delta$ x = 10 $\upmu$m (**a**), 20 $\upmu$m (**b**), 30 $\upmu$m (**c**) and 40 $\upmu$m (**d**). Epidermis (E) and dermis (D) are labeled in (**a**), with a clear distinction between the two layers. The spiral structure of sweat ducts is visible as a bright structure in (**a**) (red arrows).

**Figure 6:**
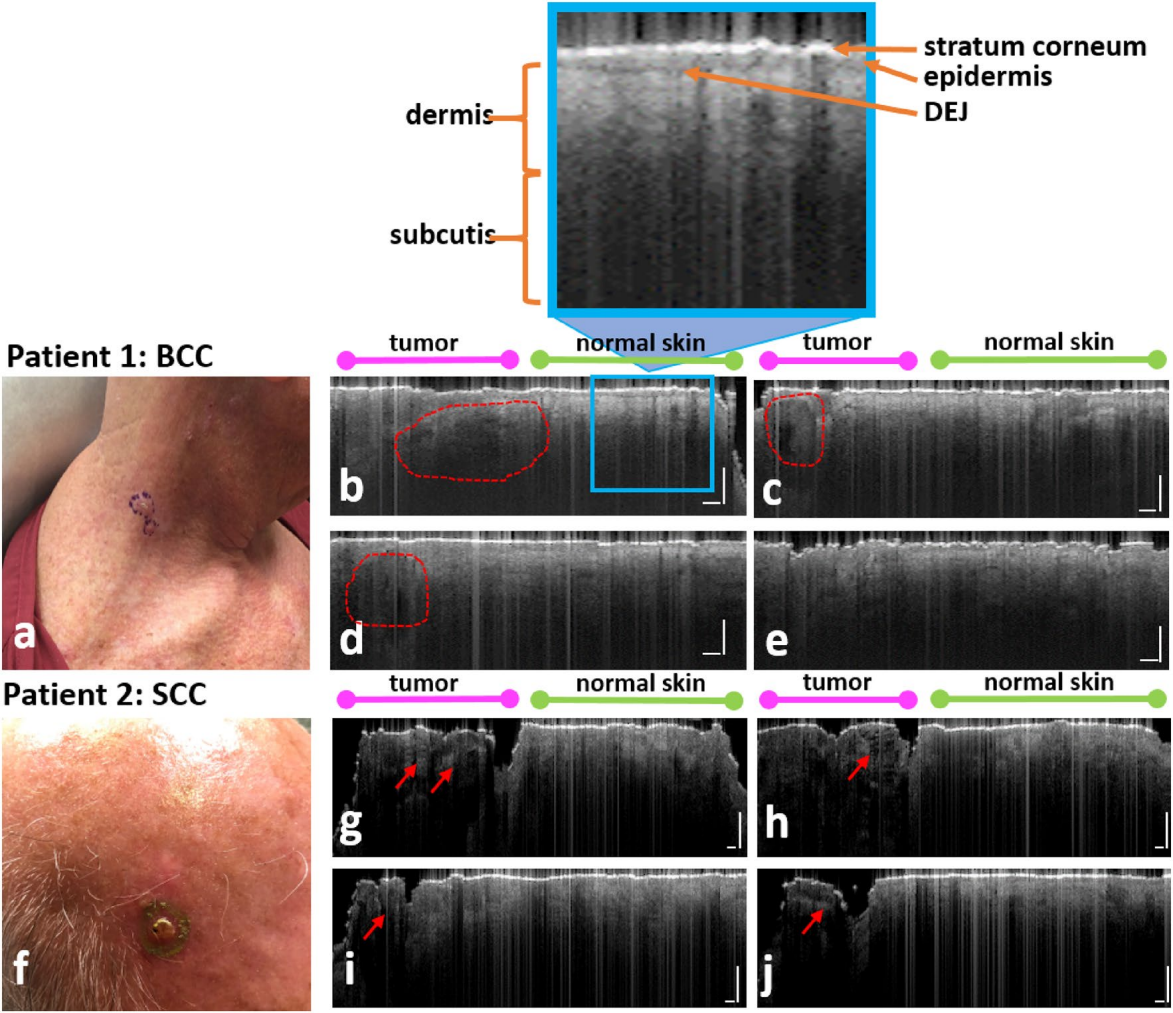
(**a**) Clinical photo of the BCC lesion on the right neck imaged in Patient 1, with (**b**–**e**) showing corresponding OCT images. (**f**) Clinical photo of the SCC lesion on the scalp imaged in Patient 2, with (**g**–**j**) showing corresponding OCT images. The left side of each scan (**b**–**e**), (**g**–**j**) represents the tumor, as can be seen by the disrupted epidermal architecture, with plug-like structures and upper dermis signal-free cavities in the BCC (areas enclosed by dashed red lines), and discrete bright regions below the surface in the SCC (red arrows). The right side of each scan represents adjacent normal skin, with more organized morphology.

**Figure 7:**
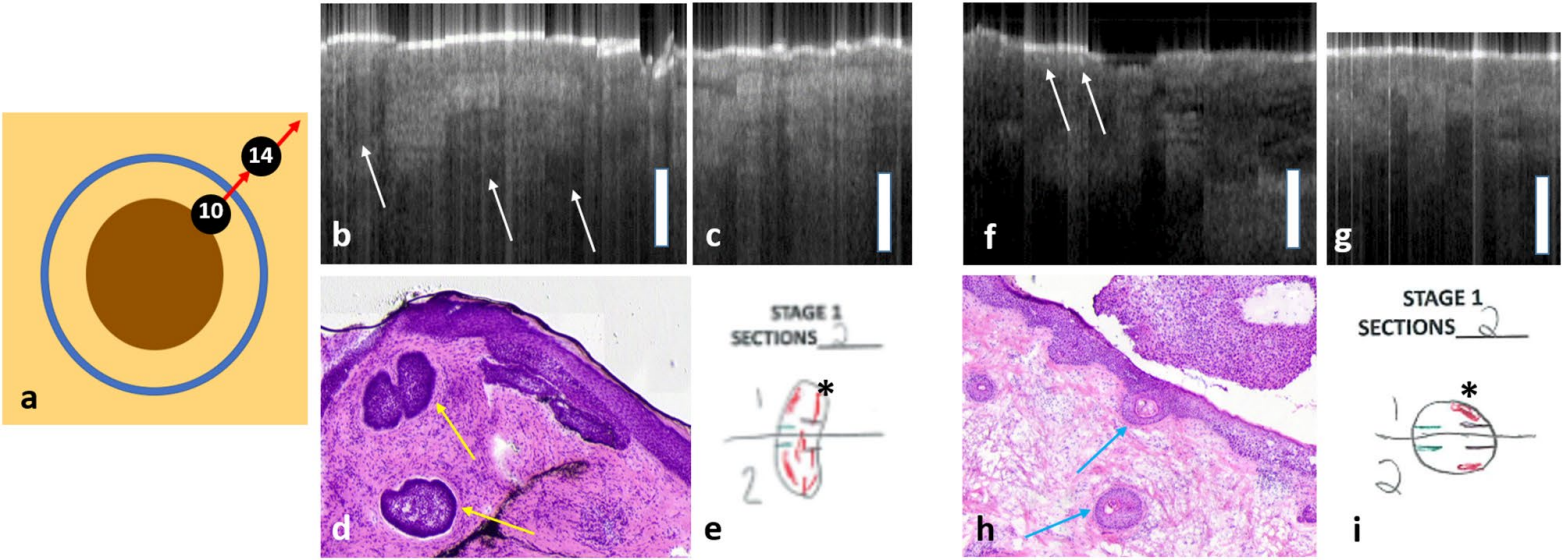
(**a**) Model demonstrating the trajectory of the scans displayed in this figure. Scan10 was done across the surgical margin and scan14 was done across adjacent normal skin. (**b**–**e**) are results obtained from Patient 1, with BCC on the right neck. (**b**) Scan10 demonstrates a positive surgical margin: dark nodular structures with a thin, dark border (white arrows). (**c**) scan14 demonstrates normal skin, with a thinner epidermis and no tumor features. (**d**) Corresponding Mohs histology demonstrates tumor islands (yellow arrows) in the region of Scan10. (**e**) Mohs histology documentation, depicting histologically positive Stage 1 margins (asterisk). (**f**–**i**) are results obtained from Patient 2, with SCC on the scalp. (**f**) scan10 demonstrates a positive surgical margin: discrete bright regions within the epidermis (white arrows). (**g**) scan14 demonstrates normal skin, with a uniform epidermis and no tumor features. (**h**) Corresponding Mohs histology demonstrates keratin pearls in the region of Scan14 (blue arrows). (**i**) Mohs histology documentation, depicting histologically positive Stage 1 margins (asterisk).

We further demonstrated that sfOCT allowed highly flexible spatial sampling to cover a region of interest with different dimension through manual scanning. We obtained images (Fig. 5) from the index finger (left hand between the proximal and distal interphalangeal joints) of a healthy volunteer, by manually scanning the single fiber OCT probe. Images in Fig. 5 were obtained by sampling 150 Ascans from distortion corrected images, at different sampling intervals ($\delta x$=10 $\upmu$m, 20 $\upmu$m, 30 $\upmu$m, 40 $\upmu$m). Notably, in Fig. 5 and subsequent figures (Figs. 6 and 7), the scale bars represent 500 $\upmu {\text{m}}$. Hence, Fig. 5a has the smallest lateral field of view (FOV), while Fig. 5d has the largest FOV. All the images in Fig. 5 clearly show epidermis (E in Fig. 5a), and dermis (D in Fig. 5a). Ascans were obtained with high spatial density in Fig. 5a, showing the spiral structure of sweat ducts^28^. With increased lateral sampling interval in Fig. 5b–d, sweat ducts remain visible, but with few structural details.

To demonstrate different signal characteristics for tumor and normal skin in the same OCT image, Fig. 6 presents results obtained from two patients (Patient 1 with BCC and Patient 2 with SCC). 2D images were obtained by manually scanning the single fiber probe across the boundary of the skin tumor, along trajectories indicated by arrows in Fig. 2b. Ascans were sampled with a large lateral interval (approximately $51\,\upmu {\text{m}}$) to achieve a sufficiently large FOV and cover the center of the tumor, tumor margin, and adjacent normal skin. Patient 1 had a BCC tumor on the right neck, as demonstrated in Fig. 6a. Figure 6b–e show the corresponding OCT images from Patient 1. Patient 2 had a SCC tumor on the scalp, as shown in Fig. 6f. The corresponding OCT images are represented in Fig. 6g–j. Each OCT scanning started from the center of the tumor, moved beyond the margin labeled by the surgeon, and ended at normal skin. Hence, the left part of each OCT scan (indicated by the magenta bars above the OCT images in Fig. 6) corresponds to the tumor. As illustrated in Fig. 6, when the probe moved to normal tissue surrounding the tumor (right part of each OCT image, indicated by the green bars), OCT signals showed a uniform epidermis, with a clear delineation representing the DEJ. This can be seen more clearly in the inset of Fig. 6b that shows a bright stratum corneum, homogenous medium grey epidermis, a darker grey DEJ representing a clear transition to the underlying light grey dermis, and dark grey-black subcutis. In each OCT image, regions corresponding to the tumor show a disrupted epidermis, losing the clear demarcation between the DEJ. Moreover, manual scanning images obtained from BCC (Fig. 6c–f) also show BCC features reported in the literature, including plug-like structures and upper dermis signal-free cavities (areas enclosed by dashed red lines)^29^ . Manual scanning images obtained from SCC (Fig. 5h–k) show SCC features reported in literature, including a highly reflective surface with discrete bright regions below the surface (red arrows)^30^.

Further identification of specific tumor features in both cases was achieved by correlating OCT scans with Mohs histological sections taken from the first stage of MMS, as well as reviewing the literature for established BCC and SCC patterns on conventional OCT (Fig. 7)^30^. For both Patient 1 and Patient 2, the top right corner of the surgical margin (approximately between 1 o’clock and 2 o’clock), was positive on histopathology from the first stage of MMS. Hence OCT scans acquired from the clinical margin and normal skin (scan10 and scan14 with the numbering of the scans consistent with Fig. 2a), between 12 o’clock and 3 o’clock, were compared to correlate with histopathology. As illustrated in Fig. 7a, scan10 was obtained across the clinically determined surgical margin, and scan14 was obtained from surrounding normal skin. Figure 7b–e and f–i show results obtained from Patient 1 and Patient 2, respectively. For Patient 1, the OCT scan obtained at the surgical margin (Fig. 7b) demonstrated dark nodular structures with a thin, dark border (white arrows) in the area of this histopathologically positive margin. This correlated to BCC tumor islands in the Mohs histopathology (Fig. 6d with yellow arrows indicating tumor islands and (e) with asterisk depicting histologically positive Stage 1 margins). For Patient 2, the OCT scan obtained from the surgical margin (Fig. 7f) demonstrated discrete bright regions within the epidermis in the area of this histologically positive margin, correlating to keratin pearls of SCC that were also seen in the Mohs histopathology (Fig. 7h). In scans of immediately adjacent normal skin taken in both cases (Fig. 7c,g), the OCT image is more organized, with prominent bright stratum corneum, medium grey uniform epidermis, and light grey dermis, with a clear demarcation between the epidermis and dermis: the DEJ.

**Figure 8:**
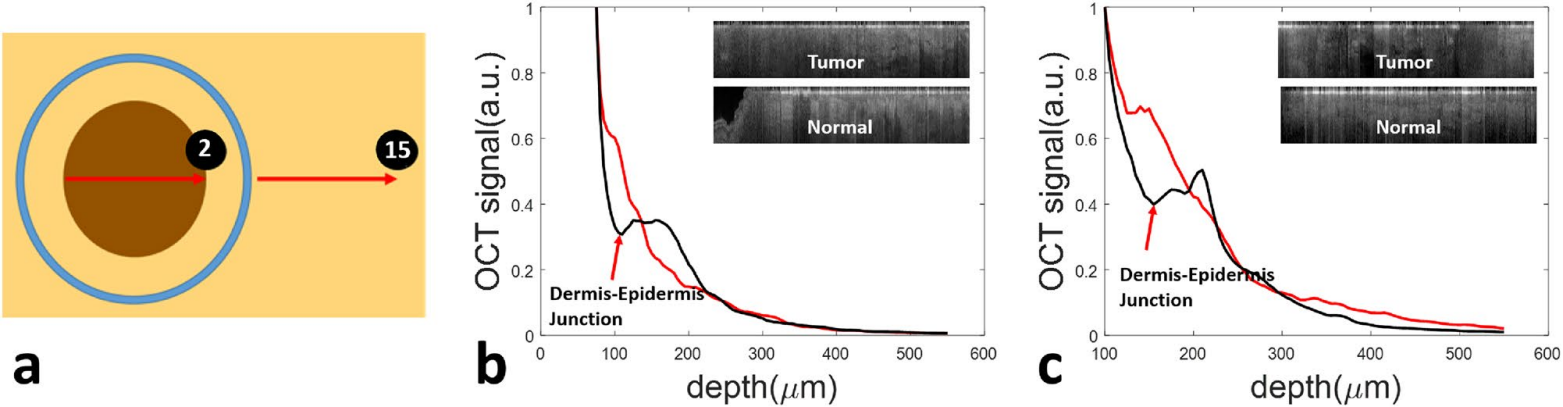
(**a**) OCT data obtained from the tumor (scan2) and adjacent normal tissue (scan15); (**b**) Depth profiles of the tumor (red) and adjacent normal skin (black) for Patient 1; (**c**) Depth profiles of the tumor (red) and adjacent normal skin (black) for Patient 2. In normal skin of both (**b**) and (**c**), the signal corresponding to the DEJ forms a depression in the black curve, which is absent in the tumor curves.

As suggested by Figs. 6 and 7, a prominent OCT feature in cancerous skin tissue is the disruption of the DEJ, while the transition between the epidermis and dermis is generally more apparent in OCT images obtained from normal skin tissue. To further demonstrate tissue differentiation capability of OCT based on DEJ detection, we compared OCT signals obtained from the center of the tumor and from the normal skin surrounding the tumor, using images obtained through manual scans along trajectories shown in Fig. 8a where the numbering of the scans is consistent with Fig. 2a. To generate a depth profile from which the DEJ could be identified, we determined the surface of the skin using the peak of individual Ascans, digitally re-aligned the Ascans to flatten the surface, and averaged the signals along the lateral scanning dimension. The flattened 2D images are illustrated as insets to Fig. 8b,c. Figure 8b shows the depth profile obtained from the tumor of Patient 1 (red) and the depth profile obtained from the normal skin of Patient 1 (black). Figure 8c shows the depth profile obtained from the tumor of Patient 2 (red) and the depth profile obtained from normal skin of Patient 2 (black). Signals obtained from normal skin (black curves in Fig. 8b,c) initially decay with a larger attenuation coefficient in epidermis, then increase to reach a peak, and decay again at a smaller attenuation coefficient in dermis. In comparison, signals obtained from skin tumors (red curve in Fig. 8b from BCC and red curve in Fig. 8c for SCC) do not show features of DEJ, suggesting disputation of DEJ in cancerous skin tissue.

## Discussion

In this study, we described and validated an OCT imaging platform based on a single fiber probe. To further justify the effectiveness of single fiber OCT in delineating skin tumor boundary, we plan to recruit more patients and conduct larger scale research. Our results qualitatively correlated histopathologically positive Mohs surgical margins with positive OCT scans (Fig. 7). Surgeons can use this tool to determine peripheral surgical margins with greater precision, through image-guided assessment that correlates to desired histopathological outcomes, while minimizing the number of required stages of MMS. Existing in vivo imaging tools, such as standard OCT and reflectance confocal microscopy, cannot pinpoint the exact area being imaged. This handheld sfOCT pen allows for more precise correlation of the image to the location.

The sfOCT system enjoys the simplicity offered by the common path interferometry and lens-less probe. The common path interferometer derives its reference power from Fresnel reflection (approximately 3.4 percent) at the fiber tip and the reference power may not correspond to an optimized sensitivity. Nevertheless, images obtained from our sfOCT system show high signal to noise ratio (SNR) and clearly visible structural features for diseased and normal skin tissues. This is because the reference light and sample light have comparable power level (in similar orders of magnitude). On the other hand, the lens-less fiber-optic probe outputs a diverging beam. Therefore, the lateral resolution of the sfOCT system degrades as imaging depth. The lateral resolution was measured to be  35 $\upmu {\text{m}}$ at an imaging depth of 1.1mm. This is comparable to the lateral resolution of a conventional OCT system using a weakly focused light beam. In summary, the sfOCT system allows effective skin imaging, despite the known limitations of a lens-less common path probe. In our previous studies, we investigated methods to optimize the reference power in a common path OCT probe and developed an ultrathin lensed fiber optic probe to achieve larger working distance and lateral resolution^31,32^. We may incorporate these novel designs for our future investigation of OCT in dermatology.

The lateral resolution of the single fiber probe can also be calculated theoretically, using the lateral profile of the Gaussian beam. For the single mode fiber used in this study (Thorlabs, SM980-5.8-125) with mode field diameter $5.8\,\upmu {\text{m}}$, the waist size of the output beam varies as depth: $w(z)=w_0 \sqrt{1+\frac{z}{{\pi {w_0}^2}/{\lambda }}}$, where $w_0=2.9\,\upmu {\text{m}}$. Considering the beam profile output from the fiber probe and the fiber’s photon collection aperture, we can estimate the full width half maximum lateral resolution to be $\delta w=107\,\upmu {\text{m}}$ ($\delta w=2w(z){ln(2)/2)}^{1/2}$). However, experimental characterization suggested a much higher lateral resolution (Fig. 3c). Moreover, our skin tissue imaging results show lateral resolution better than  $100\,\upmu {\text{m}}$ because of its capability to reveal the spiral morphology of a sweat duct (Fig. 5a)^28^. This deviation may derive from the fact the OCT detects ballistic photons and majority of detected signal comes from specular reflection. As a result, the light beam reflected from the sample is geometrically offset from the fiber core in addition the beam diffraction. This further reduces the lateral spreading of OCT signal and results in a higher lateral resolution compared to the beam width. The use of a high NA fiber or GRIN lens may lead to an improved lateral resolution, compared to a single fiber probe. Another factor that limits the quality of OCT image obtained from a single fiber probe is the reference power level. The amount of light reflected from the fiber tip cannot be precisely controlled and may affect the OCT system performance. Nevertheless, our experimental measurement of signal to noise ratio (SNR) showed an SNR larger than 60dB at small imaging depth and approximately 40dB SNR at a 2mm imaging depth, suggesting it was feasible to obtain high quality signals using the single fiber probe. The use of High NA fiber or GRIN lens may address the limitations of a single fiber probe. We previously investigated fiber optic probe with different designs to optimize the beam profile and the reference power^31–33^. Despite its limitations, a flat tip single fiber probe is more robust compared to those novel probes and was adopted in our in vivo experiments. In the future, we will investigate the application of these probes in skin imaging.

Traditional interpretation of OCT scans requires additional expertise from the human reader. In this study, we demonstrated the ability to quantitatively represent tumor vs. normal skin features, using OCT signals derived from the DEJ as an example (8) OCT images obtained by the single fiber imager not only show features in the epidermis, but also show features in the dermis. This will open vast opportunity for tumor identification. We recently demonstrated the potential of artificial intelligence to assist in determining presence or absence of a tumor, making this device accessible by all Mohs surgeons regardless of OCT experience^34^. In the future, we will develop a tool that labels tumor boundary at patient’s skin with visible marker, by integrating the OCT imager described in this manuscript, our AI algorithms and a motor actuated tissue marking mechanism. With precisely labeled tumor boundary, the surgeon is likely to achieve a negative margin after one excision stage and statistically reduce the number of stages required in Mohs surgery.

## Supplementary information

Supplementary Video.
